# Novel variants of the ATRX gene identified in MYCN non-amplified Neuroblastoma in Brazilian patients

**DOI:** 10.1016/j.clinsp.2025.100652

**Published:** 2025-04-25

**Authors:** Thamiris Magalhães Gimenez, Vanessa Pretes Peralta, Ricardo Rodrigues Giorgi, Karina Morikawa, Carolina Camargo Vince, Nathalia Halley, Sheila Aparecida Siqueira, Israel Bendit, Lilian Maria Cristofani, Vicente Odone Filho, Estela Maria Novak

**Affiliations:** a Instituto do Cancer do Estado de Sao Paulo (ICESP/ITACI), São Paulo, Brazil; b Laboratório de Investigação Médica em Pediatria Clínica -Lim-36.Instituto da Criança. Hospital de Clínicas, Faculdade de Medicina, Universidade de São Paulo (HC/FMUSP), São Paulo, Brazil; c Laboratório de Investigação Médica em Patogênese e Terapia dirigida em Onco-Imuno-Hematologia (Lim 31). Departamento de Hematologia, Hospital das Clínicas, Faculdade de Medicina, Universidade de São Paulo (HCFMUSP). São Paulo, São Paulo, Brazil; d Hospital Israelita Albert Einstein (HIAE). São Paulo, Brazil; e Divisão de Patologia, Hospital das Clínicas (HCFMUSP), Faculdade de Medicina, Universidade de São Paulo, São Paulo, Brazil; f Departamento de Pediatria, Faculdade de Medicina FMUSP, Universidade de São Paulo, São Paulo, SP, Brazil; g Fundação Pró-Sangue Hemocentro de São Paulo, Departamento de Genética Molecular e Biotecnologia. São Paulo, Brazil

**Keywords:** Advanced MYCN Non-Amplified Neuroblastoma, Older Age at Diagnosis, ATRX Loss-of-Function

## Abstract

**Background:**

Neuroblastoma is one of the most common extracranial solid tumors in children and it frequently displays high heterogeneity throughout the course of the disease. It has previously been described those changes in the *ATRX* gene (Alpha Thalassemia/Mental Retardation, X-linked) are the most common recurring events in the indolent clinical subtype (∼30 %) of *MYCN* amplified neuroblastoma. There is no effective treatment for this type of neuroblastoma, which is associated with overall poor survival. On the other hand, few studies have detected an association between high-risk (stage IV) non-amplified *MYCN* neuroblastoma patients and mutant *ATRX*.

**Methods:**

In this study, 37 tumor samples from Brazilian patients with stages I to IV *MYCN* non-amplified neuroblastoma, according to the International Neuroblastoma Staging System (INSS), were analyzed using the panel Oncomine™ Childhood Cancer Research Assay.

**Results:**

The authors found two older children (NB1 and NB2) with advanced *MYCN* non-amplified neuroblastoma carried each one of the two following novel nonsense *ATRX* variants (p.Gln1670* or p.Glu1984*). These variants created a stop codon in the helicase domain of the *ATRX* gene*,* leading to ATRX loss-of-function. These mutations were confirmed by Sanger sequencing and the protein loss-of-function was confirmed by immunohistochemistry. The finding of these heterozygous mutations in two patients with *MYCN* non-amplified neuroblastoma deserves further investigation. Thus, the authors analyzed each of these cases to better understand how these mutations may be related to disease severity and prognosis.

**Conclusion:**

ATRX loss-of-function from p.Gln1670* or p.Glu1984* mutations turn *MYCN* non-amplified neuroblastoma more aggressive and similar to what is seen in *MYCN* amplified neuroblastoma. This information may help clinical decision-making and facilitate establishing an accurate prognosis for patients with *MYCN* non-amplified neuroblastoma.

## Highlights

• Inactivation of the *ATRX* tumor suppressor in *MYCN* non-amplified neuroblastoma.
• ATRX mutations have been associated with complete loss of protein expression.
• *ATRX* mutations in neuroblastoma tend to have a progressive course of disease.
• *ATRX* mutations are associated with age at diagnosis in patients with neuroblastoma.

## Introduction

Neuroblastoma (NB) is a childhood malignancy that arises from precursor cells of the sympathetic nervous system and the adrenal medulla.^1^ It is a highly heterogeneous disease, with clinical behavior ranging from spontaneous regression to drug resistance and metastasis ultimately resulting in death.^2^^,^^3^ The median age at diagnosis for neuroblastoma is 18-months, and 40 % of the patients are younger than 1 year at diagnosis.^2^^,^^4^

The prognosis of the advanced form of the disease in older patients (over 18-months of age) is very poor with a 5-year overall survival of approximately 20 %, despite more aggressive therapies.^2^ Neuroblastoma can show many different chromosomal abnormalities, the most noticeable ones being related to the *MYCN* proto-oncogene. Its amplification is clearly associated with an independent unfavorable prognosis.^5^

However, *MYCN* amplification and *MYCN* non-amplified mechanisms and how they are potentially associated with more aggressive behavior remain to be clarified.^5^, ^6^, ^7^ *MYCN*-amplified tumors make up about 40 % of high-risk NBs, indicating that 60 % of high-risk NBs are *MYCN* non-amplified tumors.^7^

Thereby, despite the extensive study of the genomic characteristics of high-risk NB including *MYCN*-amplified tumors, genomic profiling of *MYCN* non-amplified NB, including low- and intermediate-risk NB, has been limited.^6^, ^7^, ^8^, ^9^

*MYCN* proto-oncogene, although representing an unequivocal component within the determination of neuroblastoma aggressiveness, is not the sole responsible for it. Other elements and interaction between these elements certainly play also a significant role in the pathogenesis of neuroblastoma.^5^, ^6^, ^7^, ^8^ Among the recurrent somatic mutations in chromatin remodelers, *ATRX* mutations are associated with high-risk disease, older age at diagnosis, and poor prognosis in *MYCN* amplified neuroblastoma.^8^, ^9^, ^10^, ^11^

Cheung et al.^10^ identified on a cohort of 40 diagnostic neuroblastoma *MYCN* amplified samples, mutations in the ATRX gene in 100 % (95 % CI 50 %‒100 %) of tumors from patients in the adolescent and young adult group (5 of 5), in 17 % (95 % CI 7 %‒36 %) of tumors from children (5 of 29), and 0 % (95 % CI 0 %‒40 %) of tumors from infants (0 of 6).

*ATRX* is crucial for the development of the nervous system, and germline mutations of *ATRX* cause developmental defects and neuronal cell death.^9^^,^^11^^,^^12^ The *ATRX* gene is located on the Xq13.3; and contains 35 exons. It is a chromatin-associated protein with a long C-terminal that pack together to form a single globular domain containing a helicase/ATP domain (encoded by exons 18–31) .^12^^,^^13^

The previous domain was formed by seven conserved “helicase” motifs found in DNA-stimulated ATPases and DNA helicases of the SNF2/SWI2 (Switching defective/Sucrose non fermenting) protein family.^12^^,^^13^ The SWI/SNF complexes act as global gene regulators, changing the chromatin structure and altering the accessibility of transcription factors to DNA in a subset of specific genes.^12^^,^^13^

ATRX is involved in a wide range of biological processes as cell cycle-dependent phosphorylation, which regulates its nuclear matrix and chromatin association and suggests its involvement in gene regulation at interphase and chromosomal segregation in mitosis.^12^^,^^13^ However, the occurrence of *ATRX* mutations can cause changes in the molecular and clinical aspects of tumor characteristics.^9^, ^10^, ^11^, ^12^

*ATRX* mutation leads to decreased ATRX protein expression, and results in tumor genome instability, higher tumor mutation burden, and thus leading to increased sensitivity to chemotherapy, radiation therapy and immunotherapy agents.^9^^,^^11^^,^^14^ It has been demonstrated that *ATRX* is frequently mutated in a variety of tumors including *MYCN* amplified neuroblastoma,^11^ gliomas,^15^ neuroendocrine neoplasms^16^ and sarcomas.^17^

However, the data on the incidence of *ATRX* mutation and clinical significance in *MYCN* non-amplified neuroblastoma is limited.^7^^,^^9^^,^^10^

Here, the authors report two novel nonsense mutations in the *ATRX* gene in two unrelated older than 18 months of age children with advanced *MYCN* non-amplified neuroblastoma. *ATRX* gene variants may play an important role in identifying more effectively those patients who will have an increased risk of developing chronic or indolent *MYCN* non-amplified neuroblastoma, and should be considered for future target therapies and therapeutic approaches.

## Material and methods

### Patients and samples

The authors analyzed a total of 37 tumor samples from patients with *MYCN* non-amplified neuroblastoma with stage I‒IV, according to the International Neuroblastoma Staging System (INSS), treated at Instituto de Tratamento de Cancer Infantil (ITACI)/Instituto da Criança/Hospital das Clínicas, University of Sao Paulo Medical School, Brazil, between 2021 and 2022 (Table 1S). All tumor samples were subjected to targeted sequencing using the Oncomine™ Childhood Cancer Research Assay (OCCRA®) panel (Thermo Fisher Scientific®, Waltham, MA, USA), followed by Sanger sequencing for validation. Fresh tumor samples were cryopreserved at the time of surgery. Most patients with stages III and IV had unfavorable histology, in contrast with those with neuroblastoma stage I. Clinical history, imaging exams, and other laboratory tests were used for case descriptions.

The study was approved by the Ethics Committee of Clinic's Hospital of São Paulo's University (CAAE: 63283316500000065). Written informed consent was provided by a parent or guardian of each child. In addition, this study is a clinical observational study, following the Strengthening the Reporting of Observational Studies in Epidemiology (STROBE) guidelines.

### DNA extraction

Genomic DNA from neuroblastoma tumor samples was extracted using DNAeasy Kit (QIAGEN®) according to the manufacturer's instructions. The DNA concentration was determined using a Thermo Scientific NanoDrop 2000® spectrophotometer (Waltham, MA, USA) and Qubit 3® fluorometer (Thermo Fisher Scientific®).

### Oncomine™ childhood cancer research assay, library preparation, and sequencing

Samples were analyzed using the Oncomine™ Childhood Cancer Research Assay (Thermo Fisher Scientific®), according to the manufacturer's protocol. DNA and RNA libraries were generated using Ion AmpliSeq Library Preparation on the Ion Chef System (Thermo Fisher Scientific®) and sequenced using the 540 chips on the Ion Torrent S5 (Thermo Fisher Scientific®). Complementary DNA (cDNA) synthesis prior to library preparation for the RNA panel was carried out using SuperScript™ VILO™ Reverse Transcriptase (Thermo Fisher). The readings obtained were aligned to the hg19/GHCh37 human reference genome on the Torrent Suite™ software version 5.2.1 (Thermo Fisher). The authors worked with readings with a minimum coverage of 2000×. The generated BAM files were analyzed using Integrative Genomics Viewer software (IGV Software, San Diego, CA, USA) (https://software.broadinstitute.org/software/igv/).

### Sanger sequencing

PCR products were purified by ExoSAP-IT® (USB, Cleveland, OH, USA) and used as *t* template for direct automated DNA sequencing. Sanger reaction mixture consisting of 1 μL of 10 μM sequencing primer (a forward PCR primer or a reverse PCR primer, Table 1), 1 μL of BigDye Terminator (v 1.1/Sequencing Standard Kit, Applied Biosystems, Foster City, CA, USA), 3.5 μL 5× buffer, and 14.5 μL of molecular grade H_2_O. Sanger sequencing was performed using an ABI3730xl DNA Analyzer (Applied Biosystems, Foster City, CA, USA). As a control, the authors used DNA samples from a healthy donor.

**Table 1:** Primer sequences.

**Primer**	**(5′‒3′)**
NB1-F	GTGATGCATATTTCAGTGGGAAT
NB1-R	TTCAACTTGCTTCTTTATGTCACTG
	
NB2-F	GGAAGTGGCAGTGACAATGA
NB2-R	GAGAGCACAGAGTAGCTTGCTT

### ATRX immunohistochemistry

Formalin-fixed, paraffin-embedded tumor samples from patients studied were used for immunohistochemistry. Formalin-fixed, paraffin-embedded tissues were cut into 4 μm-thick sections and immunostained with a polyclonal antibody against *ATRX* (1:250; #HPA0001906 Sigma-Aldrich, St. Louis, MO) by using heat-induced epitope retrieval and the ultra-View Universal DAB Detection Kit, 760‒500 (Ventana, Tucson, AZ, USA) on a fully automated (Ventana BenchMark XT instrument) according to the manufacturer's specifications. Nuclei were visualized by counterstaining with Hematoxylin II (Cat. n° 790–2208, Ventana) for 4 min and incubation with a bluing reagent (Cat. n° 760–2037, Ventana) for 4 min. A sample of patients with stage IV (INSS) lacking *ATRX* mutation p.Q1670* or p.Glu 1984* analyzed by OCCRA® showing ATRX positive protein expression was used as neuroblastoma control to *MYCN* non-amplified neuroblastoma patients studied. Slides were examined by two independent pathologists blinded to patients’ characteristics and outcomes.

## Results

### Cases description

#### Case NB1

A 4-year-old boy was diagnosed with INSS high-risk stage 4, *MYCN* non-amplified neuroblastoma originating from the adrenal medulla, with bone, bone marrow, and lymph node metastasis. Persistent bone marrow tumor infiltration was observed after induction treatment with TOPO—CTX-VCR-DOXO—CBDCA-ETO. After four courses of rescue therapy with temozolomide + irinotecan, the bone marrow still showed neuroblastoma infiltration. He was submitted to one course of dinutuximab, followed by isotopic therapy with I^131^-MIBG, reaching tumoral clearance.

**Fig. 1:**
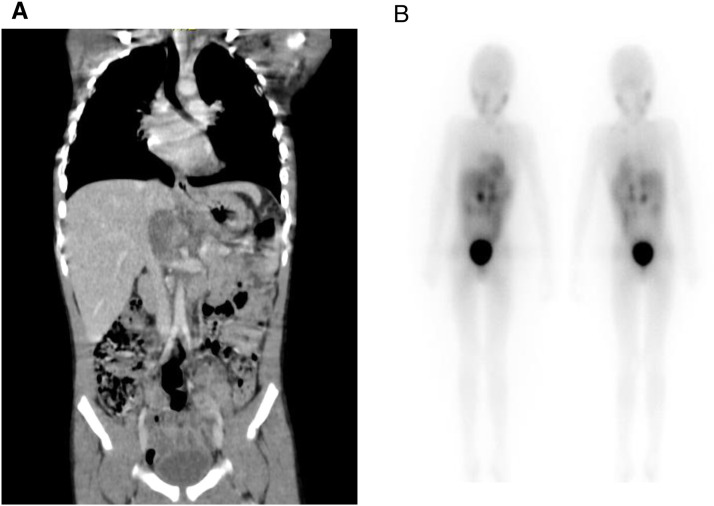
(A) Abdominal tomography after induction with chemotherapy and before the second evaluation, showing a solid retroperitoneal lesion, hypovascularized and with foci of calcification in between, close to the hepatic hilum. (B) MIBG¹³¹ scintigraphy reveals increased uptake in the left infraclavicular lymph node, retroperitoneal region near the periceliac area, hepatic hilum, para-aortic region, inter-aortocaval area, and paracaval lymph nodes.

The patient was submitted to a conditioning regimen with busulfan and melphalan and then underwent an autologous bone marrow transplant. To control abdominal residual disease, the patient also underwent abdominal radiotherapy with 24 Gy. Currently, the patient is using retinoids as metronomic chemotherapy. He is alive 30+ months after diagnosis (Fig. 1).

#### Case NB2

A 6-year-old female was diagnosed with INSS high-risk stage 4 neuroblastoma with primary adrenal mass and bone metastases that included skullcap, lymph nodes, and the bone marrow, unfavorable histology, *MYCN* non-amplified, and *ALK* with no detectable abnormal mutations. She was submitted to four cycles of chemotherapy with TOPO+CTX, alternating with more than four cycles of DOXO+VCR+CBDCA+ETO. Before surgery, a biopsy and immunohistochemistry assay revealed that the bone marrow still had infiltration, and thus rescue cycles with irinotecan and temozolomide were performed.

After six cycles, the bone marrow was finally clear. A second surgery was then performed for total resection of the abdominal mass. The patient was later referred for isotopic therapy using I131 to eliminate undesired cells in vivo before progressing to the collection of cells from the peripheral blood for autologous bone marrow transplantation. For treatment consolidation, a conditioning regimen with busulfan and melphalan was done, followed by an autologous bone marrow transplant.

After medullary recovery, the authors performed abdominal (24 Gy) and skull prophylactic radiotherapy (1800cGy) with retinoids. The patient still presented thrombocytopenia six months following the Peripheral Blood Stem Cell Transplantation (PBSCT). Patient evaluation revealed recurrence of the disease, with the bone marrow infiltrated by neuroblastoma and progression of abdominal/lymph node disease.

Rescue chemotherapy using topotecan and cyclophosphamide was initiated while waiting for the administration of anti-GD2 immunotherapy.

After three cycles of TOPO+CTX, she started irinotecan + temozolomide + betadinutuximab, with reassessments at every two cycles. Due to significant medullary toxicity intense thrombocytopenia, and the fact that bone marrow remission was achieved after six cycles, treatment was continued with betadinutuximab only.

After two cycles of betadinutuximab, the patient showed negative scintigraphy with MIBG123 and PET CT with non-uptake FDG. However, a lesion in the frontal central nervous system suggestive of bleeding was initially observed. Subsequent imaging after four cycles indicated no change in the lesion.

On the eighth cycle of immunotherapy, she had a convulsion. The MRI revealed a progression of the lesion in the frontal region. She was submitted to resection of the lesion, which was performed with free margins and without clinical complications. The lesion was compatible with a neuroblastoma. Reevaluations were conducted using MIBG 123 and PET scans with FDG, both showing no uptake, along with a bone marrow biopsy revealing no infiltration.

She was submitted to skull radiotherapy, with 2 Gy plus temozolomide, followed by beta-dinutuximab alone to complete 12 cycles.

**Fig. 2:**
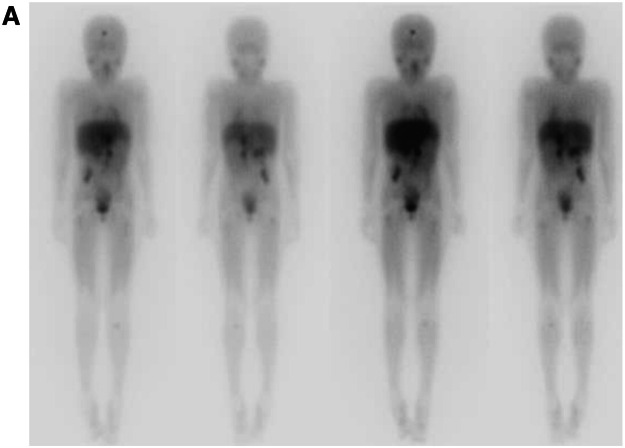
(A) Scintigraphy using MIBG¹³¹ shows accumulation of radiopharmaceutical built-up in various areas: near the frontal bone, affecting the right renal vessels, celiac trunk, and superior mesenteric artery, extending caudally in the retroperitoneum. Nodules are present behind the cecum, alongside the psoas major and right iliacus muscles, as well as in the metaphyseal areas of the left humerus and tibia. Additionally, nodules are observed in the proximal metadiaphyseal areas of the femurs and left ischium. The images on the left depict the anterior and posterior MIBG-131 pre-infusion scans, while those on the right show the same angles 48 h after drug infusion.

After 4 months of radiotherapy, the patient developed a new brain lesion. A palliative treatment was initiated. The patient died after four years of diagnosis (Fig. 2).

### Molecular analysis

The authors identified nonsense *ATRX* mutations in 2 of our 37 (5.4 %) patients studied (Table 1). Both patients were over 18 months at diagnosis, similar to patients described by Cheung et al.^10^ DNA samples from patients’ tumors were extracted and analyzed by panel Oncomine™ Childhood Cancer Research Assay as described in Supplementary Material and Methods.

**Fig. 3:**
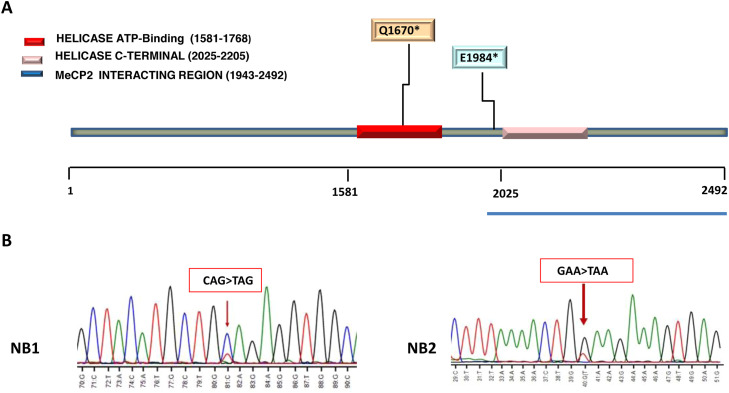
(A) Heterozygous ATRX variants. Localization of nonsense mutation involved in helicase domain are reported in the figure key. Helicase domain organization of the ATRX protein are depicted using different colors. (B) Sanger sequencing confirming stop-gain mutation in ATRX. The ATRX variants identified in patient NB1: c.5008C>T (p.Q1670*), creates a stop codon (CAG>TAG) at position 1670 (arrow). In patient NB2, variant c.5950 G>T (p.Glu1984*), creates a stop codon (GAA>TAA) at position 1984 in exon 25 (arrow). DNA samples from a healthy donor was used as control.

The two ATRX mutated cases (NB1 and NB2) were localized in the helicase domain of ATRX protein (Fig. 3A).

Patient NB1 was heterozygous for mutation c.5008C>T (p.Q1670*/Gln1670*) in exon 19 that creating a stop codon (CAG>TAG) at position 1670. The allele frequency of variant p.Gln1670* in this patient was 23.30 %.

Patient NB2 carried the heterozygous mutation c.5950 G>T (p.E1984*/Glu1984*) that similarly to the NB1 patient's mutation, it creates a stop codon (GAA>TAA) at position 1984 in exon 25. The allele frequency of Glu1984* variant in this case was 44.59 % (Table 2). Sanger sequencing was done to confirm the nonsense mutations c.5008C>T (p.Q1670* and c.5950 G>T (p.E1984*), as shown in Fig 3.

**Table 2:** Somatic ATRX mutation identified by Oncomine™ childhood cancer research assay.

**Cases**	**Chr**	**Chr Position**	**Ref Allele**	**Alt Alelle**	**Function Codon**	**EXON**	**Location**	**DNA change**	**AA change**	**Allele Frequency**	Oncomine GeneClass	**Oncomine Variant Class**
NB 1	X	76,888,821	G	A	TAG	19	Exonic	c.5008 C>T	p.Gln1670*	23.30 %	Loss-of-function	Truncating
NB2	X	76,854,886	C	A	TAA	25	Exonic	c.5950 G>T	p.Glu1984*	44.95 %	Loss-of-function	Truncating

Chr, Chromosome; AA, Amino Acids.

**Fig. 4:**
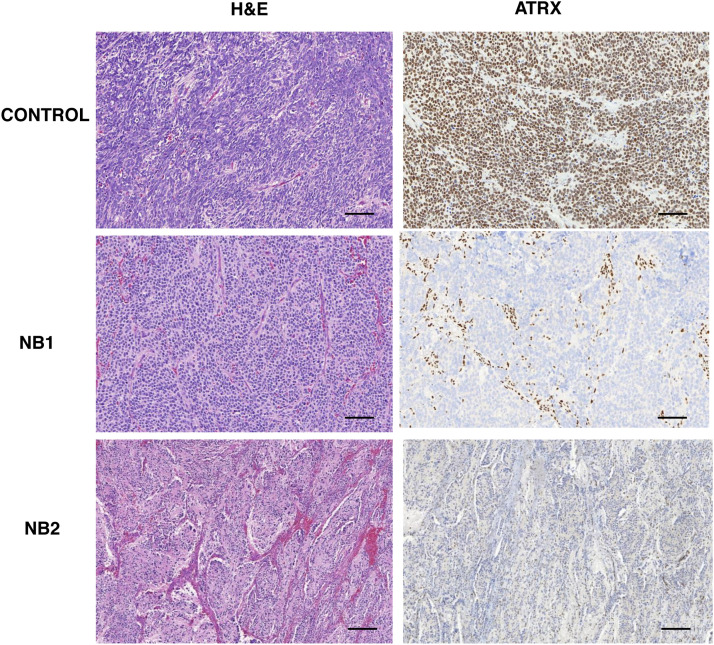
Immunohistochemical reactivity of the ATRX protein. Representative MYCN non-amplified neuroblastoma sample lacking ATRX mutation used as positive control show nuclear expression of ATRX in neoplastic cells (brown). NB1 and NB2 patients’ samples show mosaic/heterogeneous staining patterns of the ATRX (NB1), but retained expression in few non-malignant cells (brown) or complete loss of the nuclear ATRX protein (NB2). H&E staining (right) and ATRX immunohistochemical (left). For all panels, original magnification ×100. Scale bar, 10 μm.

These mutations may also affect the normal ATRX protein expression, as observed by immunohistochemistry (Fig. 4) showing mosaic/ heterogeneous staining patterns of the ATRX (NB1) or complete loss of the nuclear ATRX protein (NB2).

## Discussion

Although in pediatric cancers the abnormalities occurring in the *ATRX* gene are most frequently point mutations, this type of mutation is not common in neuroblastoma.^9^, ^10^, ^11^ Previous studies showed 23 nonsense/frameshift ATRX mutations in neuroblastoma, whereas 59 cases were described for all other pediatric cancers.^11^

However, *ATRX* mutations in neuroblastomas are often in-frame deletions that remove approximately half of the amino terminus of the protein.^9^ In other cancers, the mutations are indels or nonsense mutations.^9^

Furthermore, the effect of *ATRX* nonsense mutations in *MYCN* non-amplified neuroblastoma has not been extensively studied in patients.

In this study, 37 tumors from *MYCN* non-amplified neuroblastoma patients were sequenced and the authors found two older children (NB1 and NB2) with advanced *MYCN* non-amplified neuroblastoma carried each one of the two following novel nonsense *ATRX* variants (p.Gln1670* or p.Glu1984*).

All two *ATRX* mutations showed reduced or absence of ATRX protein production in *MYCN* non-amplified neuroblastoma, as observed by immunohistochemistry and it may have affected the normal ATRX protein expression. In a previous study, *ATRX* mutations were associated with loss of the nuclear ATRX protein in neuroblastoma.^10^

The variants p.Gln1670* or p.Glu1984* identified in patients are within the helicase domain of ATRX in the C-terminal helicase/adenosine triphosphatase domain (ATPase). ATRX has two primary functional domains, the N-terminal ADD domain and the C-terminal helicase/adenosine Triphosphatase domain (ATPase) where most mutations are found.^13^^,^^18^^,^^19^

Recently, van Gerven et al.^11^ demonstrated in 127 neuroblastoma tumor samples that *ATRX* missense mutations were predominantly present in the helicase domain and that they are predicted to disturb protein function, whereas nonsense mutations are randomly distributed across the gene and could result in the absence of ATRX protein production.

The helicase domain is required for the DNA translocation activity of the ATRX.^21^, ^22^, ^23^ Defects in the helicase domain may lead to disruption of histone variant H3.3 incorporation or other remodeling processes, such as removal of G-quadruplexes or R-loops.^21^^,^^22^

Furthermore, helicase domain mutations cause DNA double-strand breaks, and multiple exon deletion leads to dysregulated protein expression and increased tumor aggressiveness.^22^, ^23^, ^24^

In addition, the variant p.E1984* is located in an important region between the Helicase ATP-binding domain at position 1581‒1768 and the Helicase C terminal domain at position 2025‒2205,^13^^,^^19^ where ATRX interacts with the Methyl-CpG binding Protein 2 (MeCP2), a master epigenetic modulator of transcription.^20^

The presence of mutations in this region may interfere with the MECP2-ATRX interaction, impacting the binding between the two proteins. This may lead to epigenetic alterations, including abnormal levels of DNA methylation.^20^

These genetic findings raise the possibility that variants p.Gln1670* or p.Glu1984* identified in two older patients can disrupt processes such as histone variant H3.3-ATRX or MECP2-ATRX interaction leading to reduced or missing expressing of the neuroblastoma ATRX protein.

In an important study, Cheung et al.^10^ suggested that inactivation of the *ATRX* pathway correlates with older age at diagnosis and may provide a molecular marker and potential therapeutic target for neuroblastoma among adolescents and young adults. It may also delineate the subset of children with neuroblastoma who have a chronic but progressive clinical course.

Taken together, the present data show that inactivation of the *ATRX* tumor-suppressor gene from p.Gln1670* or p.Glu1984* variants may be correlated with high-risk disease and poor prognosis of these two older children.

ATRX loss-of-function in *MYCN* amplified neuroblastoma was shown to be synthetically lethal in mouse models and cell lines.^9^^,^^25^ George et al.^25^ showed in neuroblastoma models that ATRX loss-of-function results in impairment of DNA damage repair by homologous recombination and impaired replication fork processivity.

Moreover, Koschmann et al.^26^ demonstrated in mouse glioblastoma that ATRX loss causes genetic instability, including both microsatellite instability and impaired telomere maintenance. It accelerated tumor growth rate and reduced median survival.

Therefore, the authors postulated that the complete loss of ATRX function in NB2 may have accelerated tumor growth and reduced median survival, uncovering the impact of ATRX loss of function in neuroblastoma proliferation, as observed in previous studies.^10^^,^^11^^,^^25^^,^^26^

In addition, Cheung et al.^10^ demonstrated in patients with neuroblastoma, that the short-term survival for the adolescent and young adult group of patients is better than among children, but the overall survival is worse. This reflects the chronic or indolent disease progression in this older age group.

As the understanding of how *ATRX* influences aggressivity in *MYCN* non-amplified neuroblastoma is similar to that found in *MYCN* amplified neuroblastoma,^10^^,^^11^ an important aspect of this will be to explore therapeutic vulnerabilities in patients with *ATRX* mutation and ATRX loss protein.

ATRX loss-of-function in glioblastoma results in a genetically unstable tumor that when left untreated is more aggressive than those with ATRX function, but the former is more responsive to double-stranded DNA-damaging agents (doxorubicin, irinotecan, and topotecan), resulting in improved overall survival.^25^

In a previous study, George et al.^25^ demonstrated that ATRX mutant cells also showed selective sensitivity to DNA-damaging agents as capacitabine and irinotecan in neuroblastoma. Thereby, the data raise the possibility that topoisomerase inhibitors (topotecan or irinotecan) might be clinically useful in neuroblastoma *MYCN* amplified or *MYCN* non-amplified patients with ATRX loss-of-function, as was observed in patient NB1.

## Conclusion

This study is a single-center observational study that involves a relatively modest sample size of enrolled patients with *MYCN* non-amplified and limited duration of follow-up. However, these results expand the mutational spectrum of the *ATRX* gene associated to poor prognosis in *MYCN* non-amplified neuroblastoma, despite a small sample. To enhance the robustness and validity of these conclusions, future studies with larger cohorts of patients will be required to determine if the variants detected in the present study may be potential candidates for prospectively identifying an accurate prognosis for chronic or indolent patients with *MYCN* non-amplified and *MYCN* amplified neuroblastoma.

## Authors' contributions

Each author has made an important scientific contribution to the study and has assisted with the drafting or revising of the manuscript.

## Conflicts of interest

The authors declare no conflicts of interest.

## Appendix Supplementary materials

Image, application 1

## References

[1] Matthay K.K., Maris J.M., Schleiermacher G., Nakagawara A., Mackall C.L., Diller L. (2016). Neuroblastoma. Nat Rev Dis Primers.

[2] Hu X., Zhou Y., Hill C., Chen K., Cheng C., Liu X. (2024). Identification of MYCN non-amplified neuroblastoma subgroups points towards molecular signatures for precision prognosis and therapy stratification. Br J Cancer.

[3] Boeva V., Louis-Brennetot C., Peltier A., Durand S., Pierre-Eugène C., Raynal V. (2017). Heterogeneity of neuroblastoma cell identity defined by transcriptional circuitries. Nat Genet.

[4] London W.B., Castleberry R.P., Matthay K.K., Look A.T., Seeger R.C., Shimada H. (2005). Evidence for an age cutoff greater than 365 days for neuroblastoma risk group stratification in the Children's Oncology Group. J Clin Oncol.

[5] Aygun N. (2018). Biological and genetic features of neuroblastoma and their clinical importance. Current Ped Rev.

[6] Lee J.W., Son M.H., Cho H.W., Ma Y.E., Yoo K.H., Sung K.W. (2018). Clinical significance of MYCN amplified in patients with high-risk neuroblastoma. Pediatr Blood Cancer.

[7] Lee E., Lee J.W., Lee B., Park K., Shim J., Yoo K.H. (2020). Genomic profile of MYCN non-amplified neuroblastoma and potential for immunotherapeutic strategies in neuroblastoma. BMC Med Genomics.

[8] Pudela C., Balyasny S., Applebaum M.A. (2020). Nervous system: embryonal tumors: neuroblastoma. Atlas Genet Cytogenet Oncol Haematol.

[9] Zeineldin M., Federico S., Chen X., Fan Y., Xu B., Stewart E. (2020). MYCN amplification and ATRX mutations are incompatible in neuroblastoma. Nat Commun.

[10] Cheung N.-K.V., Zhang J., Lu C., Parker M., Bahrami A., Tickoo S.K. (2012). St Jude Children's Research Hospital-Washington University Pediatric Cancer Genome Project. Association of age at diagnosis and genetic mutations in patients with neuroblastoma. JAMA.

[11] van Gerven M.R., Bozsaky E., Matser Y.A.H., Vosseberg J., Taschner-Mandl S., Koster J. (2022). Mutational spectrum of ATRX aberrations in neuroblastoma and associated patient and tumor characteristics. Cancer Sci.

[12] Shahbazi Z., Rostami G., Hamid M. (2022). New heritable ATRX mutation identified by whole exome sequencing and review. Egypt J Med Human Genetics.

[13] Valenzuela M., Amato R., Sgura A., Antoccia A., Berardinelli F. (2021). The multiple facets of ATRX protein. Cancers (Basel).

[14] Xie Y., Wang H., Wang S., Feng Y., Feng Y., Fan S. (2021). Clinicopathological significance of ATRX expression in nasopharyngeal carcinoma patients: a retrospective study. J Cancer.

[15] Nandakumar P., Mansouri A., Das S. (2017). The role of ATRX in glioma biology. Front Oncol.

[16] Pekmezci M., Rice T., Molinaro A.M., Walsh K.M., Decker P.A., Hansen H. (2017). Adult infiltrating gliomas with WHO 2016 integrated diagnosis: additional prognostic roles of ATRX and TERT. Acta Neuropathol.

[17] van den Bent M.J., Weller M., Wen P.Y., Kros J.M., Aldape K., Chang S. (2017). A clinical perspective on the 2016 WHO brain tumor classification and routine molecular diagnostics. Neuro Oncol.

[18] Stabile M., Colavito D., Del Giudice E., Rispoli A.F., Ingenito M.C., Anna K., Naumova A.K. (2020). A novel exomal ATRX mutation with preferential transmission to offspring: a case report and review of the literature for transmission ratio distortion in ATRX families. Mol Med Rep.

[19] Darmusey L., Gaelle Perot G., Thebault N., Le Guellec S., Desplat N., Gaston L. (2021). ATRX alteration contributes to tumor growth and immune escape in pleomorphic sarcomas. Cancers (Basel).

[20] Nan X., Hou J., Maclean A., Nasir J., Lafuente M.J., Shu X. (2007). Interaction between chromatin proteins MECP2 and ATRX is disrupted by mutations that cause inherited mental retardation. Proc Natl Acad Sci USA.

[21] Mitson M., Kelley L.A., Sternberg M.J.E., Higgs D.R., Gibbons R.J. (2011). Functional significance of mutations in the Snf2 domain of ATRX. Hum Mol Genet.

[22] Yuan K., Tang Y., Ding Z., Peng L., Zeng J., Wu H. (2024). Mutant ATRX: pathogenesis of ATRX syndrome and cancer. Front Mol Biosci.

[23] Wu Y., Brosh R. (2010). Helicase-inactivating mutations as a basis for dominant negative phenotypes. Cell Cycle.

[24] Brabant A., Stan R., Ellis N. (2000). DNA helicases, genomic instability, and human genetic disease. Annu Rev Genomics Hum Genet.

[25] George S., Lorenzi F., King D., Hartlieb S., Campbell J., Pemberton H. (2020). Therapeutic vulnerabilities in the DNA damage response for the treatment of ATRX mutant neuroblastoma. EBioMedicine.

[26] Koschmann C., Calinescu A.A., Nunez F.J. (2016). ATRX loss promotes tumor growth and impairs nonhomologous end joining DNA repair in glioma. Sci Transl Med.

