# Supplementary material for: Novel variants of the ATRX gene identified in MYCN non-amplified Neuroblastoma in Brazilian patients
Source: Clinics (Sao Paulo). 2025 Apr 25;80:100652. doi: 10.1016/j.clinsp.2025.100652 (PMC12060459; doi:10.1016/j.clinsp.2025.100652)
Supplement: Supplementary file 1 [file mmc1.docx]

**CLINICS-D-24-01076_Supplementary Material**

**Table S1** Clinical characteristics of 37 patients *MYCN* non amplified.

| **Clinical Characteristics** |  |
| --- | --- |
| **Median age at initial diagnosis, months** | 42.3 months |
| **Gender, n (%)** |  |
| Male | 17 (46%) |
| Female | 20 (54%) |
|  |  |
| **Age at diagnosis, n (%)** |  |
| ≤ 18 months | 7 (19%) |
| ≥ 18 months | 30(81%) |
|  |  |
| **Primary site, n (%)** |  |
| Abdomen | 26 (70.3%) |
| Other | 11 (29.7%) |
|  |  |
| **Stage (INSS), n (%)** |  |
| I‒II | 6 (16%) |
| III‒IV | 31 (84%) |
|  |  |
| **Clinical background, n (%)** |  |
| Relapsed | 16 (43.2%) |
| Non Relapsed | 21 (56.8%) |
|  |  |
| **Outcome, n (%)** |  |
| DOD | 9 (24.3%) |
| CR | 4 (10.8%) |
| **In treatment** | 24 (64.9%) |

INSS, International Neuroblastoma Staging System; DOD, Dead of Disease; CR, Complete Remission according to INRC.
